# Proteomic analysis of whole blood to investigate the therapeutic effects of nervonic acid on cerebral ischemia-reperfusion injury in rats

**DOI:** 10.3389/fcell.2025.1546073

**Published:** 2025-01-28

**Authors:** Qingqing Li, Fengrong Zhang, Xianyu Li, Qing Wang

**Affiliations:** ^1^ School of Life Sciences, Beijing University of Chinese Medicine, Beijing, China; ^2^ Beijing Key Laboratory of Traditional Chinese Medicine Basic Research on Prevention and Treatment for Major Diseases, Experimental Research Center, China Academy of Chinese Medical Sciences, Beijing, China

**Keywords:** whole blood proteome, cerebral ischemia-reperfusion injury, nervonic acid, ginkgo biloba extract, mechanism

## Abstract

**Introduction:**

Blood proteomics offers a powerful approach for identifying disease-specific biomarkers. However, no reliable blood markers are currently available for the diagnosis stroke. Nervonic acid (NA), a vital long-chain monounsaturated fatty acid found in mammalian nervous tissue, shows promising therapeutic potential in neurological disorders. This study aimed to develop a reliable methodology for whole blood proteomics to identify early warning biomarkers and evaluate drug treatment efficacy.

**Methods:**

After modeling via the classic thread embolization method, whole blood samples were collected from the rats. Morphological assessments of brain tissue indicated that NA significantly mitigated brain and neuronal damage in rats. The differential protein expression profile was analyzed using Liquid Chromatography—Tandem Mass Spectrometry (LC-MS/MS) whole blood proteomics.

**Results:**

Gene Ontology (GO) analysis revealed that, compared to ginkgo biloba extract (EGb), the proteins differentially expressed under NA intervention were predominantly involved in oxidative stress response and calcium-dependent adhesion processes. Key targets of NA in the treatment of middle cerebral artery occlusion (MCAO) models included ENO1, STAT3, NME2, VCL, and CCT3.

**Discussion:**

This whole blood proteomic approach provides a comprehensive understanding of protein profiles associated with disease states, offering valuable insights into potential therapeutic targets and enabling the evaluation of NA and EGb intervention efficacy. Our findings underscore the protective effects of NA against cerebral ischemia-reperfusion injury and highlight its potential as a treatment for stroke.

## Introduction

Strokes have a significant impact on the mortality and disability rates of adults in China (Report on stroke prevention and treatment in China Writing Group, 2020). The Global Burden of Disease (GBD) database reports that acute ischemic stroke accounts for 69.6%–72.8% of newly diagnosed stroke cases in China (Huo and Gao, 2023). However, there is currently a lack of reliable blood biomarkers to characterize ischemia-reperfusion injury following ischemia. With advancements in mass spectrometry and liquid chromatography, proteomics has enabled rapid and precise identification of disease-associated biomarkers, offering an improved foundation for early diagnosis and novel drug discovery. The application of proteomics in various biological samples, such as brain tissue, astrocytes, and urine, has yielded valuable insights into potential therapeutic targets for cardiovascular diseases (Wang J. et al., 2023; Yuan et al., 2023; Zeng et al., 2023; Zhang D. et al., 2024; Zhou J. et al., 2024; Zhang et al., n.d.). Previous studies have demonstrated that plasma proteomics can effectively identify protein markers in diverse immune cells, rendering them valuable targets for the diagnosis and treatment of atherosclerosis (Manta et al., 2022). The use of blood-derived antibodies is considered to be suitable for the rapid diagnosis of stroke (O’Connell et al., 2019), indicating that blood may serve as a fundamental source for expedited diagnostic procedures. Driven by advancements in liquid biopsy technology, blood proteins have emerged as a highly informative source of biomarkers for disease diagnosis and monitoring, surpassing the utility of tissue proteomics. Therefore, the establishment of whole-blood proteomic methodology is crucial to assess blood protein expression profiles in cerebral ischemia-reperfusion models, greatly contributing to research on stroke development and drug effects evaluation.

Reperfusion therapy is considered one of the most efficacious treatments for stroke; however, the occurrence of ineffective reperfusion remains a challenge (Fisher and Savitz, 2022). Consequently, the integration of pharmacological agents with reperfusion therapy has emerged as a promising approach to enhance the cure rates (Savitz et al., 2017). The generation of harmful free radicals and activation of immune cells are critical mechanisms that trigger cerebral ischemic-reperfusion injury (Nie et al., 2023). A diverse range of neuroprotective drugs (inhibiting glutamate-mediated signal transduction, calcium signal transduction, free radicals, or inflammation) have been investigated in an attempt to extend the window for thrombolytic therapy, but have confronted issues such as the inability to replicate the efficacy of the drugs in Phase III clinical trials and pronounced side effects (Savitz and Fisher, 2007). Therefore, the precise identification of drug targets within complex biological networks is essential in the development of drugs for the treatment of brain ischemia-reperfusion injury.

Nervonic acid (NA), a vital long-chain monounsaturated fatty acid found in mammalian nervous tissue, plays a vital role in brain development and neuronal biogenesis (Li et al., 2019). NA has been shown to alleviate the axon loss and functional impairment in oligodendrocyte myelination by activating blood-brain barrier cells, promoting the expression of insulin-like growth factor-1 (IGF-1), ciliary neurotrophic factor (CNTF), and brain-derived neurotrophic factor (BDNF). These factors collectively enhance myelin synthesis and provide a protective barrier for neuronal cells (Piatek et al., 2022). Additionally, NA exhibits significant anti-inflammatory effect by reducing inflammatory responses in the brain, liver, and gastrointestinal tract (Wang X. et al., 2023). Its anti-inflammatory mechanisms predominantly involve modulation of multiple pro-inflammatory signaling pathways, notably downregulating tumor necrosis factor-alpha (TNF-α) and nuclear factor kappa B (NF-κB) signaling pathways. In rat model of Alzheimer’s disease, the administration of NA has been shown to effectively reduce inflammation, leading to a decrease in the deposition level of amyloid beta peptide (Aβ) and abnormal phosphorylation of tau protein (Chen et al., 2024a). However, the mechanism by which NA treats ischemic-reperfusion injury remains unclear, necessitating further elucidation of its specific target of action.

In this study, we introduced a whole-blood proteomics approach utilizing feature enrichment technology combined with liquid chromatography-tandem mass spectrometry (LC-MS/MS) to dynamically characterize protein expression in whole blood samples under simulated disease conditions. By comparing the therapeutic effects of NA and ginkgo biloba extract (EGb) on the middle cerebral artery occlusion (MCAO) model, we identified differentially expressed proteins (DEPs) associated with the disease state and therapeutic interventions. Additionally, bioinformatics analysis was performed to highlight the advantages of whole-blood proteomics in evaluating both MACO models and drug characteristics. This approach established a comprehensive protein expression profile for rat whole blood, providing a robust foundation for future methodological advancements and clinical drug evaluation.

## Materials and methods

### Animal model construction

Male Sprague-Dawley (SD) rats, aged 6–8 weeks and weighing (200 ± 2) g, were procured from Beijing Vitoply Experimental Animal Technology Co., Ltd. (Certificate number: SCXK (Jing)2016-0001). The animal facility strictly regulated the environmental conditions to ensure optimal living standards for the rats, with a precisely maintained 12-hour light/dark cycle, indoor temperatures ranging between 22°C and 26°C, and relative humidity kept within the range of 40%–50%. The experimental protocol has been approved by the Institutional Animal Ethics Committee of China Academy of Chinese Medicine (No. ERCCACMS20-16-2002).

The MCAO model was established using the classic thread ligation method (Longa et al., 1989) and the rats were induced with 1% pentobarbital sodium anesthesia. The branches of the external carotid artery were isolated, dissected, and coagulated. The internal carotid arteries were carefully separated from the nearby vagal nerve, and the pterygopalatine artery was tied off. A thread plug was inserted into the common carotid artery and internal carotid artery until it encountered resistance at the aortic bifurcation, where it was then tightly tied. After performing layered suturing of the skin at the trauma site, the bolus wire was removed after 1.5 h to achieve reperfusion. The Sham group underwent an identical surgical procedure, with the exception of the MCAO occlusion.

After modeling, the surviving rats were randomly assigned to four groups: Sham, Model, NA (480 mg/kg, irrigation) and EGb (20 mg/kg, irrigation) (Dr. Schupp Pharma GmbH, Germany, batch number: H20090296). Sham and Model groups received an equivalent volume of sodium carboxymethylcellulose instead of the drug. Blood was collected from the abdominal aorta using a syringe containing an anticoagulant. The rat brain tissue was collected and stored at −80°C, and then immediately fixed in a 4% paraformaldehyde solution for straining.

### Neurological function scoring

After the rats regained consciousness, they were scored according to the established Longa method (Liu et al., 2020). The score of 0 represents normal neural function, with no apparent neurological deficits in the rats. The score of 4 indicates that the rats is either in a state of near death or already dead. Scores of 1–3 suggest that the rats have varying degrees of neurological deficits.

### Nissl staining

After the rat brain tissue was fixed with 4% paraformaldehyde, it was embedded in paraffin and sliced into sections. Histological section was successively treated with xylene, ethanol of varying concentrations, and distilled water, and then immersed in 1% pyrogallol for staining. Histological sections were washed with distilled water, divided into color by 70% alcohol, and dehydrated in ethanol of varying concentrations. After dehydration, histological sections became transparent after being treated with xylene, and were sealed with Di-n-butyl phthalate dissolved in xylene (DPX). Finally, the processed sections were observed under an optical microscope (Eclipse CI-L, Nikon, Tokyo, Japan) and quantitatively analyzed using ImageJ 1.8 (National Institutes of Health, Maryland, United States).

### TTC staining

After the drug administration, the rats were anesthetized. Brain tissue was extracted, quickly frozen for at −20°C 10 min, and then removed. The tissue was sliced into approximately 2 mm thick sections. The brain tissue sections were incubated in TTC staining solution and kept in the dark at 37°C for 30 min. The tissue sections were then fixed in a fixative solution containing φ_B_ = 4% paraformaldehyde for 30 min. The proportion of cerebral infarction was calculated, using ImageJ (National Institutes of Health, Maryland, United States), based on the formula “Cerebral infarction rate = area of infarcted region/total tissue area × 100%.”

### Preparation of whole blood protein samples

Blood proteins were enriched using a blood proteomics assay kit, with specific details available in the literature (Li et al., 2023; Zhang et al., 2024c). In summary, the sample was incubated at room temperature with the binding buffer (50 mM Tris, 10 mM EDTA) and magnetic polystyrene beads. After incubation, the sample was transferred to a centrifuge tube and adsorbed on a magnetic stand. Then,the beads were suspended in binding buffer again and adsorbed on a magnetic stand after being subjected to an ionic vortex. The supernatant was discarded after three washing operations using Wash Buffer and adsorption on a magnetic stand. The precipitate was then incubated with Lysis Buffer-1, Lysis Buffer-2, and Lysis Buffer-3 in sequence at 95°C for 10 min and then for 2 h at room temperature with trypsin. After enzymatic digestion, the precipitate was washed with Column Wash Buffer 1, Column Wash Buffer 2, and Column Wash Buffer 3, and the supernatant was collected after adsorption by the magnetic stand and discarding the washing fluid.

### LC-MS/MS analysis

The analysis was carried out by utilizing an Orbitrap Fusion Lumos Tribrid mass spectrometer (Thermo Fisher Scientific, United States) outfitted with a nanospray ion source and a nanoflow high-performance liquid chromatography (EASY-nLC 1000, Thermo Fisher Scientific, United States) (parameters are presented in Table 1). The samples were separated on an Ultimate XB-C18 3 μm column (75 μm × 100 mm, Welch Materials, United States). The mobile phase consisted of buffer A (99.5% H_2_O and 0.5% formic acid) and buffer B (99.5% acetonitrile and 0.5% formic acid), with a linear gradient of buffer B from φ_B_ = 3% to φ_B_ = 100%. The peptide mixture was separated. The identification results were searched using data-independent acquisition neural networks (DIA-NN) (parameters are listed in Table 1), and the peptide identification false discovery rate (FDR) was set to 1%. The unlabeled quantitative analysis of whole blood protein was performed using peak area method.

**TABLE 1:** Parameters of liquid chromatograph-mass spectrometer.

Parameter name	Parameter
Gradient Flow Rate	0.35 mL min^-1^
Electrospray Voltage	2.0 kV
Resolution	MS1: 70,000; MS2: 17,500
AGC Target	MS1: 3 × 10⁶; MS2: 1 × 10⁶
Maximum Injection Time	MS1: 20 ms; MS2: 60 ms
Scan Range	300 ∼ 1,400 m/z
False Positive Rate (FDR)	1.0%
restriction mode	trypsin (complete)
number of missing interfaces	1
minimum peptide length	7
maximum number of peptide modifications	3
ion mass error tolerance	10 ppm; 0.02 Da
fixed modificatio	cysteine iodoacetamidation (carbamidomethyl/+57.021 Da)
variable modification	methionine oxidation (oxidation/+15.995 Da) and N-acetylation (acetyl/+42.011 Da)

### Bioinformatics analysis

Proteins with an extremely low abundance or those undetectable in over 60% of the samples were excluded from DIA-NN. The *t-test* was employed to compare the protein expression between every two groups, and the differential analysis based on limma (one-way analysis of variance) was utilized to compare the protein expression levels among multiple groups. The data processing website is (https://www.omicsolution.com/wkomics/main/). Proteins were considered differentially expressed if *P* < 0.05. Proteins that were significantly upregulated or downregulated, defined by fold changes greater than 0.5 or less than −0.5, respectively, were selected from the total DEPs. After comparing the full-blood proteomics data with the disease protein atlas database (www.proteinatlas.org), the reliability of the data was confirmed. Subsequently, we utilized the bioinformatics analysis website (https://www.bioinformatics.com.cn) to generate the principal component analysis (PCA) and a heatmap to analyze the inter-group heterogeneity and quality changes of these DEPs. Using gene ontology (GO) annotation, the DEPs were studied and analyzed for their participation in biological processes, subcellular localization, molecular functions, protein-protein interaction networks and protein structural domains using DAVID Bioinformatics Resources (https://david.ncifcrf.gov) and STRING analysis software (https://cn.string-db.org/). Furthermore, the correlations between molecular functions and proteins, as well as protein-protein interactions, were further examined and visualized by using the Cytoscape 3.10.2 software.

## Results

### NA improves the neurological injury and brain tissue damage induced by MCAO in rat

**FIGURE 1:**
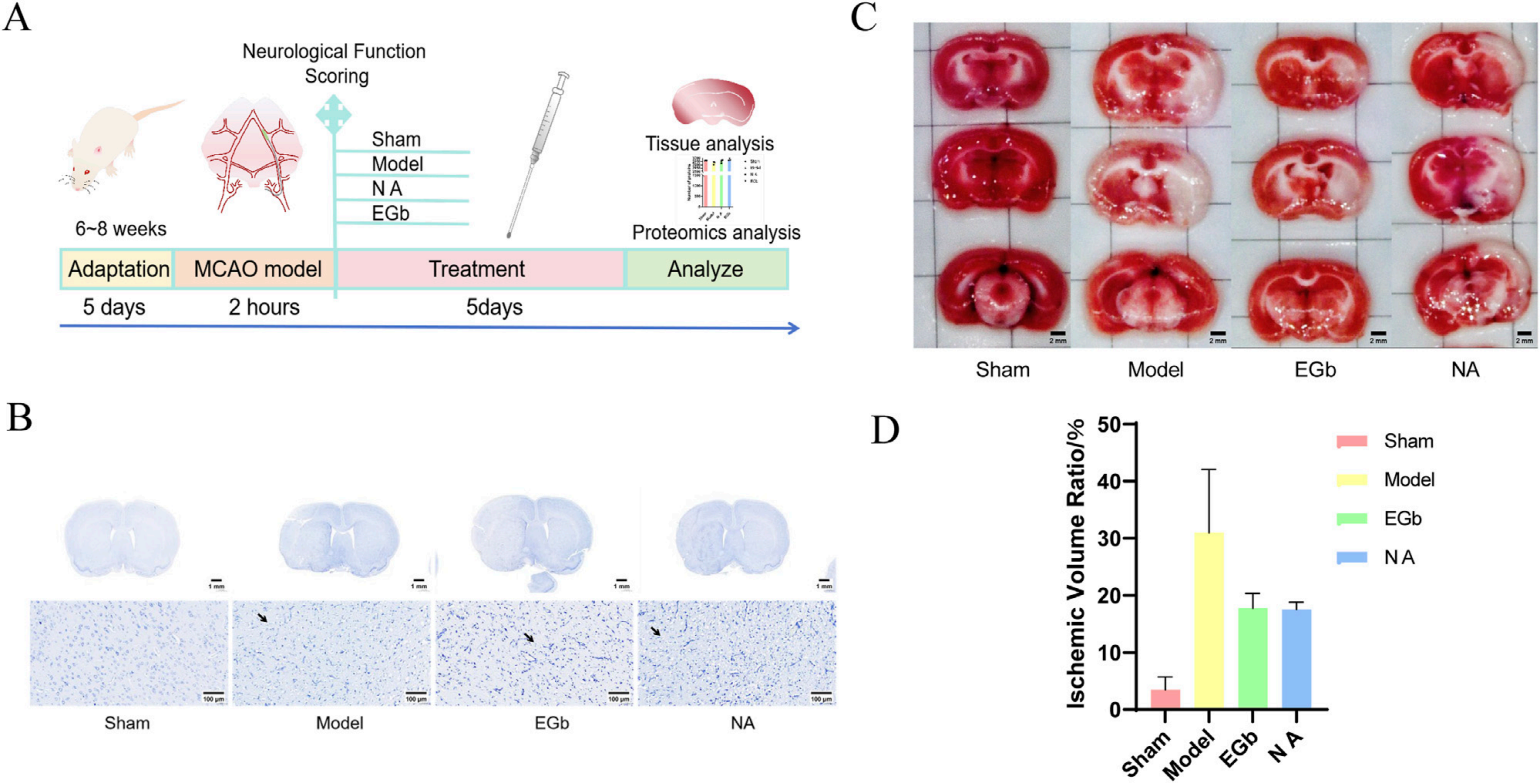
NA demonstrated substantial protection against injury caused by cerebral ischemic stroke. **(A)** Schematic protocol of the experiment design. **(B)** Nissl staining of brain tissue. The black arrows indicate the absence of Nissl bodies in the neurons within the infarcted side of the brain tissue. The NA and EGb groups exhibited a significant improvement in neuronal damage. **(C)** TTC staining of brain tissue. The red portion indicates the normal brain tissue, and the white area represents the infarction region. The NA and EGb groups ameliorated the condition of cerebral infarction. **(D)** The proportion of infarct area to the total brain volume in rats. The area of cerebral infarction in the TTC staining map was calculated by means of ImageJ software, further suggesting that the NA and EGb groups ameliorated the situation of cerebral infarction.

To validate the neuroprotective effects of NA, we assessed the impact of a 5-day NA treatment on neurological deficits and cerebral infarction in rats subjected to cerebral ischemia (Figure 1A). EGb has been shown to ameliorate oxidative stress responses and modulate the immune system, making it a potential therapeutic approach for ischaemic stroke (Liu et al., 2019; Hui et al., 2023; Zang et al., 2023), serving as the positive control. As shown in Table 2, after 5 days of treatment, the neurological function score of the Sham group (0.00 ± 0.00) was significantly lower than that of the Model group (1.44 ± 0.73, *P* < 0.01), reflecting better neurological function in the Sham group. Similarly, the scores in the NA (0.78 ± 0.67, *P* < 0.05) and EGb (0.71 ± 0.76, *P* < 0.05) groups were also significantly lower compared to the Model group. These results indicate that both NA and EGb effectively ameliorated ischemia-reperfusion injury-induced neurological impairments in rats.

**TABLE 2:** Neurological function scores in each group after 2 and 5 h.

Group	2 h score	5 h score	N
Sham	0 ± 0	0 ± 0	8
Model	1.78 ± 0.44	1.44 ± 0.73^##^	9
NA	1.78 ± 0.44	0.78 ± 0.67*	9
EGb	1.71 ± 0.49	0.71 ± 0.76*	7

Note: ^#^Compared with the Sham group,^#^ *P* < 0.05,^##^ *P* < 0.01; *Compared with the Model group,*P < 0.05,**P < 0.01.

The Nissl staining results showed a marked reduction in the number of neurons in the Model group compared to the Sham group, accompanied by cellular damage and unclear Nissl body structures. In contrast, both the NA and EGb groups exhibited higher numbers of neurons than that in the Model group, with intact cell structures and clear Nissl body structures (Figure 1B).

The TTC staining showed that the area of brain infarction was less than 3.50% in the Sham group, while the Model group displayed distinct white regions compared to the Sham group. The ratio of cerebral infarction volume was as high as 30.96%, indicating that ischemia-reperfusion has a significant injury effect on the brain. In comparation to the Model group, the NA and the EGb groups both demonstrated significant protective effects against reperfusion-induced cerebral infarction, reflecting their positive impact in mitigating brain tissue damage (Figures 1C, D).

### Methodological validation of the whole blood proteome

**FIGURE 2:**
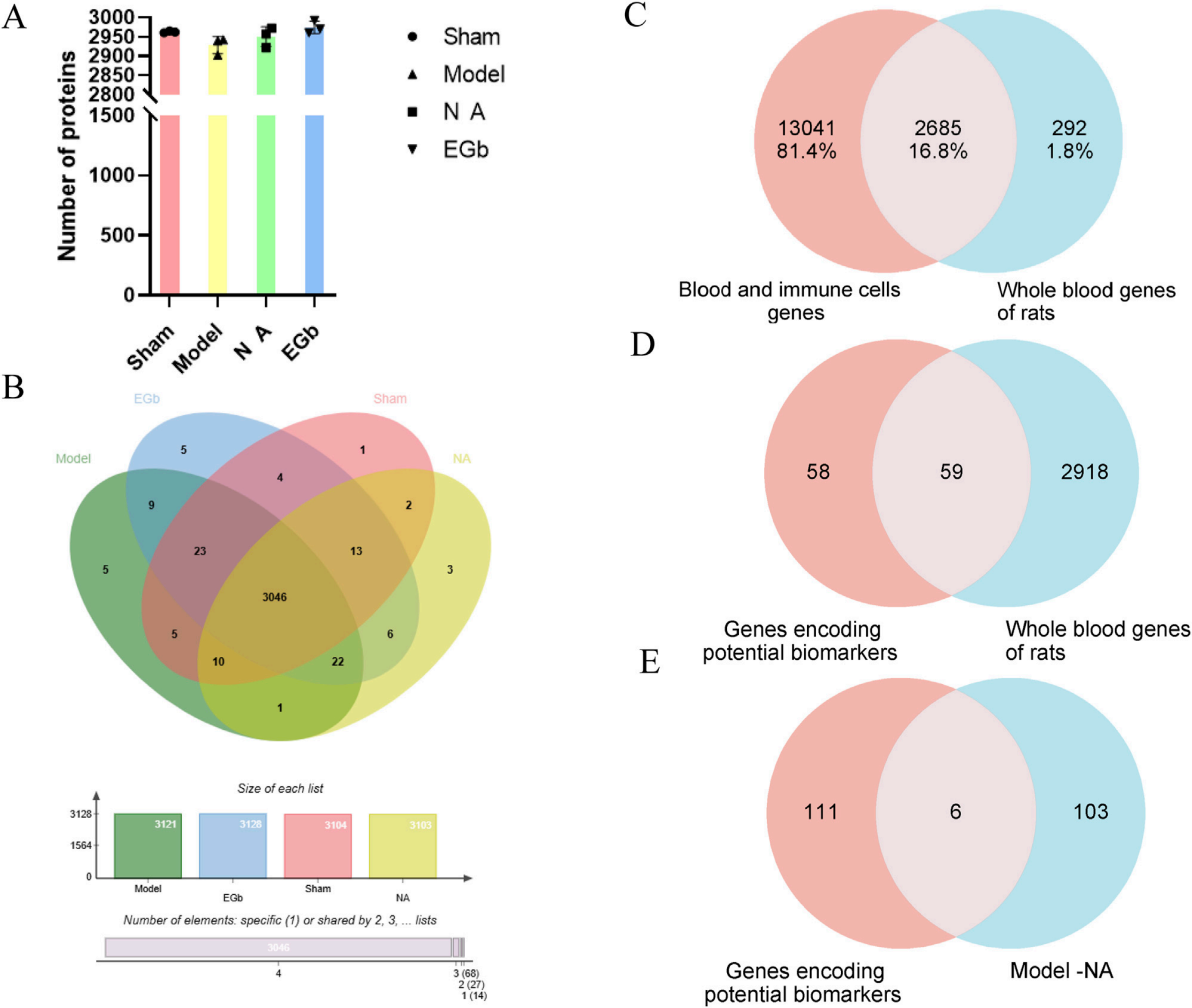
Methodological evaluation of the rat whole blood proteome. **(A)**: Histogram of the number of proteins detected in each sample. **(B)**: Venn plot between four proteomes. **(C)**: Comparison of the detected rat whole blood genome with disease-associated blood and immune cell genomes. The genomic data highly overlap with human disease genes and exhibit validity. **(D)**: Comparison of the genes encoding potential biomarkers with whole blood genes of rats. **(E)**: Comparison of the genes encoding potential biomarkers with DEPs between Model-NA groups. The results demonstrated a high degree of reproducibility and reliability in the analysis of the whole blood proteome.

To verify the reliability and data validity of the established whole blood proteome method, three samples were randomly selected from each of the four groups for inter-group coefficient of variation analysis. The results indicated that the dispersion of protein numbers among the groups was minimal, suggesting that the method exhibited a high level of reproducibility (Figure 2A). After data preprocessing, we compared the number of proteins detected in the Sham, Model, NA and EGb groups. Among the 3046 proteins detected in all four groups, there was a protein overlap rate of 97%, indicating that the method has high reliability (Figure 2B).

The disease-related blood and immune cell proteome data were retrieved from the Protein Atlas (www.proteinatlas.org) and compared with the blood protein data obtained in this study. The results revealed that more than 90% of the proteins detected in the blood had corresponding genes in the Protein Atlas database, indicating the identification results are reliable and highlighting the advantage of blood proteome identification (Figure 2C).

We also summarized the proteins that have been identified as potential markers of ischemic-reperfusion injury in previous studies (Malicek et al., 2021; Hochrainer and Yang, 2022), including blood, brain tissue, and cerebrospinal fluid samples (Table 3). These proteins are mainly associated with neuroinflammation, oxidative stress response, angiogenesis, coagulation response, and immune regulation. Compared with the proteome data in this study, over 50% of the potentially disease markers were detected, with six proteins (Corresponding gene name: SERPINA1, ENO1, PGK1, MAPK1, PRDX2, SOD1) showing significant changes in the NA group (Figures 2D, E). Among these proteins, SERPINA1, PRDX2 and SOD1 are all associated with anti-inflammatory and oxidative stress response, which are indicative of the primary effects of NA.

**TABLE 3:** Potential biomarkers of ischemic-reperfusion injury in previous literature.

Biomarkers	Sample type
GADD45G, catenin δ−2	Blood
DRP2	Brain tissue
SPNA-2 Fragment	
HSC70, HSP60, HSP90, HSPA5, HSP10, HSP70	
thioredoxin, peroxiredoxin - 2, UCH - L1	
tropomodulin - 2, calmodulin - 1, superoxide dismutase - 1, synapsin - 1, MDH2, stathmin, apolipoprotein - A1	
α−2 - macroglobulin, albumin, apolipoprotein - A1, SERPINA3K, transferrin	
cathepsin, LAMP2, complement C3, C - reactive protein, albumin, fibrinogens, annexin - 2, transferrin, α−1 - antitrypsin	
annexin - 3	
apolipoprotein - E, α−2 - macroglobulin, albumin, SERPINA3K, apolipoprotein - A1, serpins, fibrinogens, C3, S100A9, transthyretin, transferrin	
apolipoprotein - E, SERPINE2, clusterin, cystatin - C	
estradiol, PEA15, PPP2A	
Stat3 - and sex-specific differentially expressed protein (ACO2, superoxide dismutase - 2, γ - enolase, α - synuclein, synapsin - 2, 14 - 3 - 3 protein β/α, profilin - 2, DRP2, Pur - α, BASP1)	
HSP70, HSP27, guanylyl cyclase, PTAFR, β - actin, muskulin - 1	
polycomb group (PcG)protein, ATPase subunits, histones, kinases, phosphatases, Ras - related proteins	
p - SNAP25	
α - synuclein, 14 - 3 - 3 protein ζ/δ, protein disulfide - isomerase	
HSP60, PEA15, PPP2A, NSE	
DRP2	
UCH - L1, peroxiredoxin - 5, IDH	
GNAO1, MAPRE, HSP60, HSP75, fumarate hydratase	
14 - 3 - 3 protein ε	
glutamate decarboxylase - 2, EAAT1, neurofilaments, MAPT, CAMK2D, Ras - related protein - 10, α - internexin	
COX5B, superoxide dismutase - 1, peroxiredoxin - 2, claudin - 11, MBP, neurofilaments	
DRP2, BAIAP2L1, profilin - 2, MAP2K1, PPIA	
PKCβ and ε Conjugated proteins (such as 49 and 39 types of proteins), DRP2, triosephosphate isomerase - 1, HSC71, GAPDH, HSP60, GRP78, 14 - 3 - 3 protein γ, Pur - α, ACO1	
PPP1α - and γ - interacting proteins (such as HSC70, DARPP32, PDE6B, VCP, GRP78, neurofilament light polypeptide, DRP2, prelamin - A/C, γ - enolase, β - actin, SIAH2)	
4E - BP2 - interacting proteins (such as HSC70, DRP2, α - enolase, UCH - L1, adenosine kinase - 1, GAPDH, phosphoglycerate kinase - 1)	
SUMO-modified proteins (such as 91 proteins, including many RNA and transcriptional regulatory factors)	
Ubiquitinated proteins (such as 272 proteins, including many synaptic regulatory factors, translation factors, and chaperone proteins)	
RNA processing-related proteins (such as TDP43, FUS, HNRNPA1, NONO, SFPQ), HSPs, ubiquitin, SUMO, eIF4, kinases PKC& CAMK2	
GFAP, albumin, C3, fibrinogens, transferrin, NSF, GDI, ARHGDIA, HSC70, gelsolin, DRP2	
SAHH2, PACN1, SRSF1, neuromodulin, PKCγ, SIRT2	
Ras - related protein - 3C, sulfotransferase - 4A1, α−1 - acid glycoprotein 1, α−1 - antitrypsin	
vimentin, annexin - 2, HSP70, MBP, ferritin, SLC25A11, GOT2, NDUFS1	
transthyretin	Biofluids (Cerebrospinal fluid, Urine, Plasma)
C3, α−2 - macroglobulin, haptoglobin, albumin, transferrin	Biofluids (Blood, Cerebrospinal fluid)
creatine kinase - β, CAMK2A, CAMK2B, CMPK	Biofluids (Cerebrospinal fluid, Blood)
H - FABP	Biofluids (Cerebrospinal fluid, Serum)
FABP, GFAP, S100B, MBP, GSTP1, peroxiredoxin - 1, S100B	Biofluids (Extracellular fluid)
CPN2, FXII, PLG, MASP1, APCS, PON1, CA1	Blood

### Principal component analysis (PCA)

**FIGURE 3:**
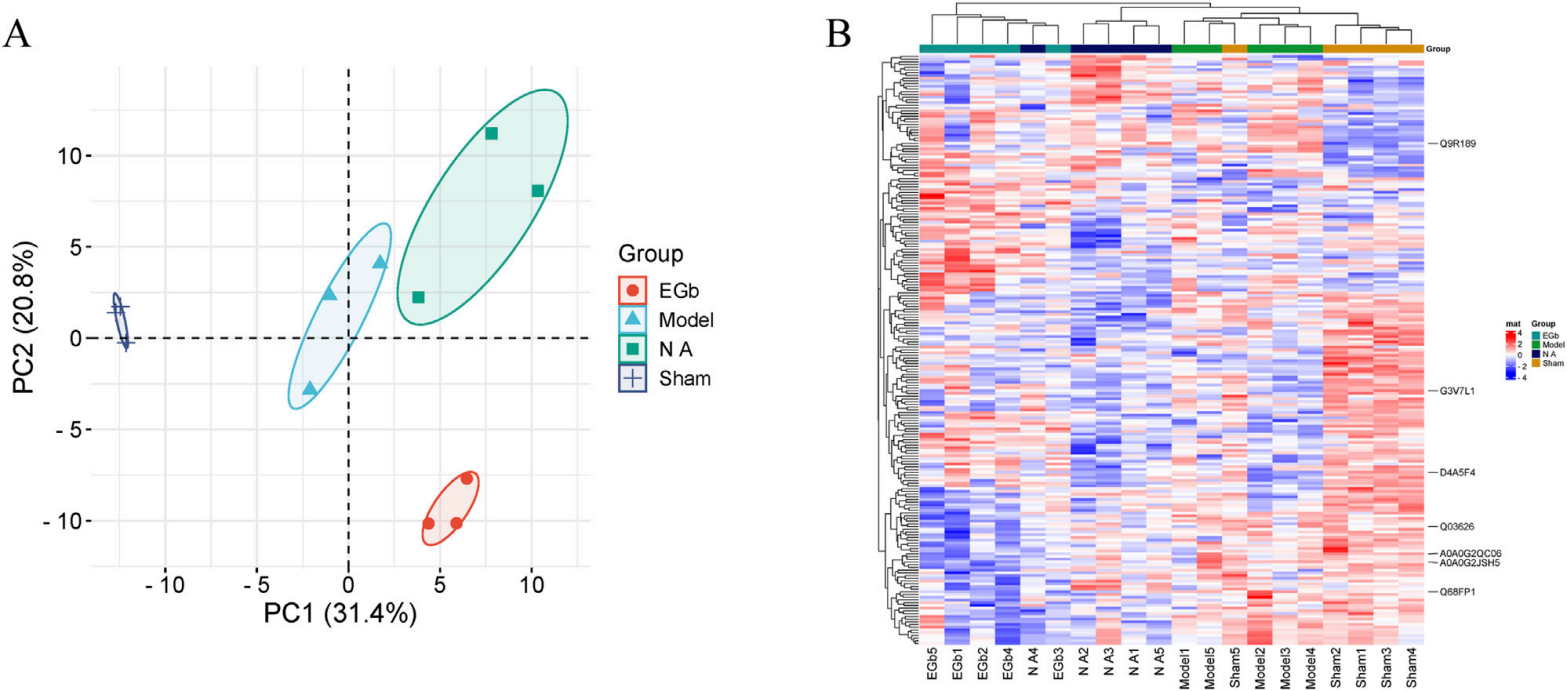
Principal component analysis (PCA) and heat map of four samples. **(A)**: PCA analysis, and dark blue represented Sham group; light blue represented Model group; green represented NA group; orange represented EGb group. **(B)**: Heat map of the DEPs and visual analysis of hierarchical clustering between Sham, Model, NA and EGb groups. The vertical axis represents DEPs, and the horizontal axis denotes groups. Red indicates high protein expression levels, while blue signifies low protein expression levels. The clustering dendrogram illustrates the degree of similarity in protein expression profiles. Branches that are closer together suggest a higher degree of similarity in protein expression between groups, whereas more distant branches indicate greater differences in protein expression. Compared with the EGb group, the protein expression in the NA group is more similar to that in the Sham group.

The expression of proteins between different groups was utilized to conduct a correlation analysis employing PCA, with ellipses representing the 95% confidence interval. The results showed a significant difference between the Sham and Model groups in the first principal component variability, while the confidence intervals of the two groups exhibited complete separation. This suggests that, compared to the Sham group, the protein expression in the model group was significantly abnormal. When comparing the EGb and NA groups to the Model group, a statistically significant difference in the variability of the first principal component was also observed, with the EGb and NA groups distinctly separated from the Model group. There was no significant difference observed in the first principal component variation between the EGb group and the NA group; however, a significant difference was noted in the second principal component variation. This indicates significant difference in protein expression between the EGb/NA and Model groups, as well as a notable difference in protein expression between the EGb group and NA group. (Figure 3A).

### Clustering analysis of DEPs

We assessed the impacts of NA on ischemia-reperfusion injury at the protein level. Most of the DEPs had obvious differences in protein content between the Sham and Model groups. Compared with the Model group, the levels of aberrant DEPs expressed in the EGb and NA groups showed some degree of recovery. The clustering analysis revealed that the NA group exhibited greater similarity to the Sham group than to the EGb group, indicating that NA may have superior therapeutic effects on ischemia-reperfusion injury. Seven prominent DEPs were marked in the Figure 3B, including G3V7L1, A0A0G2JSH5, Q03626, A0A0G2QC06, Q68FP1, Q9R189, D4A5F4 (corresponding gene names: UTRN, ALB, MUG1, TF, GSN, UNC13D, CRACD).

### GO enrichment analysis of DEPs

To acquire a deeper understanding of the functional characteristics exhibited by DEPs across different samples, we conducted Gene Ontology (GO) enrichment analysis, which systematically compares the distinctions between four groups. A total DEPs were mapped for biological processes (BP), cellular components (CC) and molecular function (MF) using GO annotations.

**FIGURE 4:**
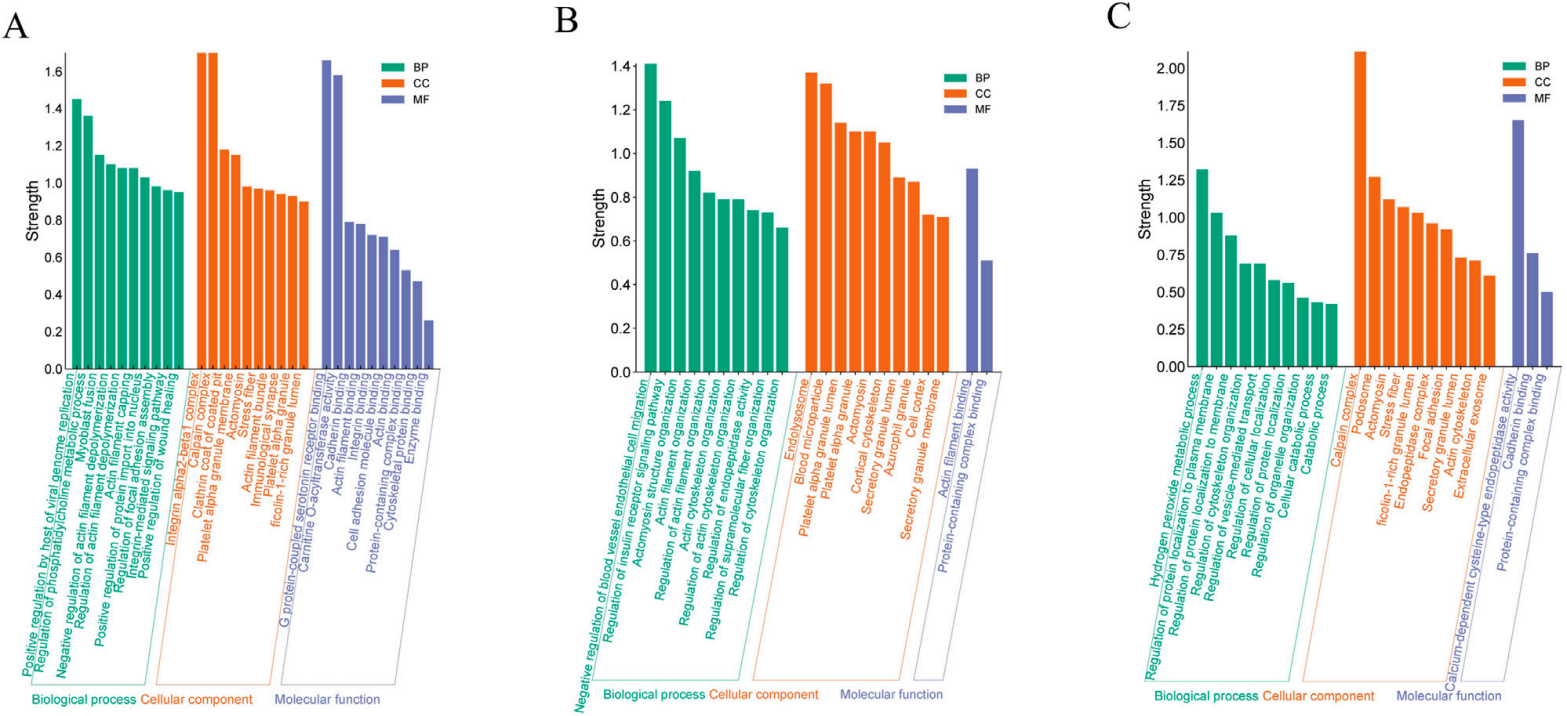
GO analysis of DEPs. **(A)** for Model and Sham; **(B)** for Model and the EGb group; **(C)** for Model and NA group. The vertical axis represents the enrichment intensity, while the horizontal axis lists specific terms under the categories of “Molecular Function,” “Cellular Component,” and “Biological Process.”

Compared with the Model and Sham groups, the DEPs were mainly concentrated in red blood cells and platelets, with subcellular localization in integrin α1-β1 complex, calpain complex, platelet α granule membrane, and ficolin-1-rich granule lumen, etc. These DEPs mainly participate in regulation of phosphatidylcholine metabolic process, regulation of actin filament depolymerization, and integrin-mediated signaling pathway. The main molecular functions include the binding of G protein-coupled serotonin receptor, carnitine O-acyltransferase activity, cadherin binding, integrin binding, cytoskeletal protein binding, and enzyme binding. These results indicate that pathological changes of ischemia-reperfusion are closely related to the immune system, oxidative stress process, and regulation of cytoskeletal structure and cell adhesion processes (Figure 4A).

Compared with the Model group, the distribution of DEPs in the NA and EGb groups exhibited significant dissimilarities (Figures 4B, C). The DEPs between the EGb and Model groups was mainly localized in the inner membrane and outer fibrous layer of blood vessels, which represent newly formed tissues following vascular injury. The results of subcellular localization showed the presence of endolysosome, blood microparticle, platelet alpha granule lumen and azurophil granule, etc. In contrast, the DEPs in the NA group were primarily located in cajal interstitial cells, and the subcellular localization analysis showed the presence of the calpain complex, ficolin-1-rich granule lumen, and endopeptidase complex, etc. There were likewise differences in the biological processes and molecular functions. The DEPs in the EGb group were mainly involved in the negative regulation of blood vessel endothelial cell migration and the regulation of endopeptidase activity, etc., which mainly had an affinity for binding to actin filaments (Figure 4B). The DEPs in the NA group primarily participated in the hydrogen peroxide metabolic process, regulation of cellular localization, etc., which displayed a calcium-dependent cysteine-type endopeptidase catalytic activity and specific binding to cadherins (Figure 4C). The findings suggest that the therapeutic function of NA in treatment of ischemic-reperfusion injury may be associated with immune system regulation, oxidative stress response, cell adhesion.

### Network between molecular function and protein interaction

**FIGURE 5:**
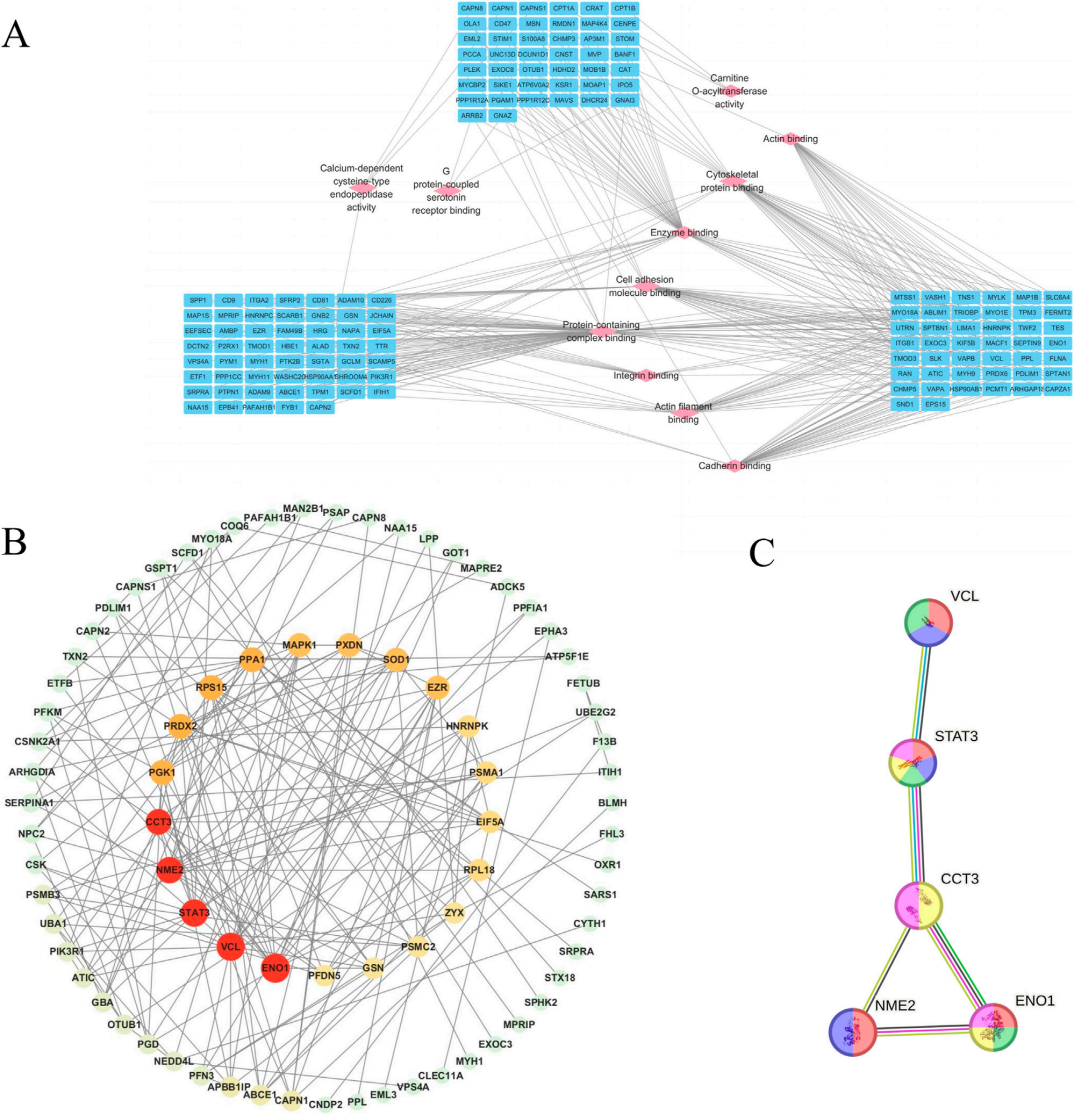
Network diagram showing molecular function-protein interactions, and key targets and the PPI network for treating cerebral ischemia-reperfusion injury in NA. **(A)**: Diagram of the interaction between molecular function and proteins. The nodes of pink diamond-shaped signify molecular functions, and the nodes of blue oval-shaped denote proteins. **(B)**: Network diagram of key targets and PPI network of core targets in the treatment of cerebral ischemia-reperfusion injury in NA. The size of the nodes is proportional to the degree value, and the red color deepens accordingly to visually present the importance of the nodes. **(C)**: Yellow indicates the oxidation-reduction process; purple indicates the cell growth, invasion, and metastasis process; red indicates the process of traumatic astrocyte injury and death; blue indicates the process of inhibiting the apoptosis of gastric cancer stem-like cells; green indicates the process of myofibroblast cell promoting cancer cell invasion and metastasis.

To further analyze the cross-regulatory relationships among DEPs in different groups, we constructed a molecular function-protein interaction network map by integrating the MF categories obtained from GO analysis. The results showed that the protein complex binding node displayed extensive connectivity with numerous DEPs (Figure 5A), highlighting the significance of regulating multiple biological processes involved in protein complex formation for effective management of cerebral ischemia-reperfusion injury. The number of DEPs with calcium-dependent cysteine-type endopeptidase activity, G protein-coupled 5-hydroxytryptamine receptor binding, and carnitine O-acyltransferase activity was less than 4 (Figure 5A). However, these three molecular functions were the most strongly enrichment in GO, underscoring their pivotal role in the treatment of cerebral ischemia-reperfusion injury. We utilized the STRING database to construct a protein-protein interaction (PPI) network map for NA-related target proteins. The findings suggest that ENO1, STAT3, NME2, VCL, and CCT3 may serve as important targets for therapy (Figures 5B, C) and are mainly involved in the process of oxidation-reduction processes, cell apoptosis, and astrocyte damage (Brentnall et al., 2012; Li et al., 2015; Levine et al., 2016; Mostek-Majewska et al., 2021a; Qi et al., 2021).

To elucidate the molecular functions of DEPs, we conducted an analysis focusing on their protein structural domains (Table 4). The spectrin repeat sequences and calponin homology domains are crucial in the DEPs of the model group, primarily contributing to the regulation of the cytoskeleton, complement and coagulation cascades, as well as actin adhesion and linkage. In the NA group, calpain III constitutes the main domain among the DEPs, which is predominantly involved in the remodeling of cytoskeletal and membrane attachments, apoptosis, and diverse signal transduction pathways. For the EGB group, serum albumin is the primary domain of the DEPs, regulating blood colloid osmotic pressure via its binding to water, cations (such as Ca^2+^, Na^+^ and K^+^), and hormones. These findings suggest that a strongly correlating between calcium ion-related signaling pathways and the therapeutic effects of NA in the context of cerebral ischemia-reperfusion injury, aligning with previous GO analysis.

**TABLE 4:** Protein domains and molecular function annotation.

Group	Domain	Cellular function	Biological process
Model-NA	Calpain_III Calpain-like thiol protease family	Signalling	Remodelling of cytoskeletal/Membrane attachments, different signal transduction pathways, and apoptosis
Model-Sham	Spectrin repeats	Binding/catalysis: actin-binding	Adherens junction, Regulation of actin cytoskeleton, Tight junction, Focal adhesion, Complement and coagulation cascades
	Calponin homology domain		Focal adhesion, Regulation of actin cytoskeleton, Adherens junction, Tight junction, MAPK signaling pathway, Fc epsilon RI signaling pathway, T cell receptor signaling pathway, B cell receptor signaling pathway, Leukocyte transendothelial migration, Natural killer cell mediated cytotoxicity
Model-EGb	Serum albumin	Serum transport proteins:it binds water, cations (such as Ca^2+^, Na^+^ and K^+^), fatty acids, hormones, bilirubin and drugs	Regulate the colloidal osmotic pressure of blood

## Discussion

Whole blood proteomics provides an extensive and integrated profile of the physiological condition by encompassing both cellular and plasma components. In contrast, plasma proteomics focuses primarily on circulating proteins, potentially missing cell-associated proteins crucial for various functions. Similarly, peripheral blood mononuclear cells (PBMCs) represent a subset of immune cells, excluding other cell types like granulocytes and erythrocytes, which limits the scope of analysis (He et al., 2019). Therefore, whole blood proteomics offers a comprehensive perspective by capturing an extensive array of proteins and offering distinct advantages in investigation of complex diseases that involve multiple physiological systems.

This study demonstrates that whole blood proteomics provides a comprehensive understanding of MCAO and drug-related protein profiles. A comparative analysis was conducted to compare markers associated with ischemia-reperfusion injury previously reported in the literature to the key targets identified in this study. Among the top five most significant biological targets identified through PPI analysis, ENO1 stood out. ENO1 has been shown to undergo substantial changes in astrocytoma tissues and is associated with the activation of NF-κB, modulation of E-cadherin expression, and the PI3K/AKT signaling pathway (Song et al., 2014). Key pathways and biological processes previously identified as potential biomarkers, such as neuroinflammation, oxidative stress, and immune regulation, were also enriched in the whole blood proteome analysis, underscoring the reliability and comprehensiveness of this method. Furthermore, our findings revealed additional proteins that may serve as biomarkers for cerebral ischemia-reperfusion injury, including STAT3 (Xu et al., 2023), NME2, VCL, and CCT3. These results highlight the potential of whole blood proteomics as a powerful tool for identifying disease markers and therapeutic targets.

Previous studies have emphasized the critical importance of inhibiting oxidative stress, neuroinflammation, and neuronal apoptosis to effectively treat cerebral ischemia-reperfusion injury (Savitz et al., 2017; Yuan et al., 2023). The pharmacodynamic effects of EGb are primarily attributed to its anti-inflammatory and antioxidant properties (Ou et al., 2009; Yuan et al., 2024) aligning with the findings of this study. Moreover, our results indicate that EGb’s therapeutic effects are closely associated with biological processes such as actin binding and endothelial cell migration, further validating the reliability of whole blood proteomic analysis. In this study, over 90% of the detected proteins were mapped to protein profiles associated with both disease-related blood and immune cells. The hematological and functional significance of these data highlights the unique advantages of this approach in comprehensively analyzing blood protein composition. It also provides valuable insights into patterns of protein alterations related to diseases and their underlying mechanisms.

Nervonic acid (NA) has been reported to exert multiple beneficial effects, including reducing inflammatory factors, regulating immune responses, and inhibiting oxidative stress (Chen et al., 2024b; Ma et al., 2024; Zeng et al., 2024). Recent studies have further revealed that NA achieves effective therapeutic outcomes in treating stroke and post-stroke depression (PSD) by modulating the brain-gut axis (Zhang et al., 2024b; Zhou H. et al., 2024). In our study, pathological analysis demonstrated that NA significantly mitigates neuronal damage, improves cerebral blood flow, and reduces cerebral infarction size. Using whole blood proteomics analysis confirmed the above findings, aligning with the observed effects on cerebral ischemia-reperfusion injury. Furthermore, it was found that NA primarily modulates cerebral ischemia-reperfusion injury through pathways involving hydrogen peroxide metabolism. Clinically, it has been observed that restoring blood flow in ischemic stroke patients can worsen brain damage, known as ischemia-reperfusion injury. Oxidative stress is a critical factor in this process. Reducing oxidative stress may significantly alleviate ischemia-reperfusion injury and subsequent pathophysiological responses (Ten and Starkov, 2012; Pawluk et al., 2024). Oxidative stress, triggered by reactive oxygen species (ROS) during cerebral ischemia, leads to cell death and ultimately brain damage, particularly following reperfusion. The reperfusion process generates reactive oxygen species, including superoxide anions, hydroxyl radicals, and nitric oxide (NO), which contribute to lipid peroxidation, inflammation, and apoptosis (Wu et al., 2020). Based on these findings, we hypothesize that the key target protein CCT3, modulated by NA, plays a critical role in attenuating oxidative stress during the therapeutic process for cerebral ischemia-reperfusion injury (Mostek-Majewska et al., 2021b). This study provides evidence that NA’s protective effects may involve oxidative stress response pathways. The results indicate that NA may alleviate oxidative stress-induced neuronal damage by enhancing the expression of antioxidant proteins and anti-inflammatory cytokines. However, further research is necessary to fully elucidate the underlying mechanisms.

The in-depth analysis of whole blood proteomics further revealed the protein alterations in cerebral ischemia-reperfusion injury. DEPs identified between the Sham and Model groups were mainly located in red blood cells and platelets, suggesting that these cellular components were the primary sites of pathology,and the regulation of cytoskeletal structure and cell adhesion may be key pathogenic process in disease development. The DEPs across the three groups (Sham vs. Model, NA vs. Model, EGb vs. Model) were implicated in biological processes of adhesion, cytoskeletal regulation and Immune system regulation. Although the therapeutic effects were observed in both the NA and EGb groups, it can be observed from the Neurological Function Score and clustering analysis results that the efficacy of the NA group surpasses that of the EGb group, our previous studies can also validate this (Zhang et al., 2024b). Our whole blood proteomic data reveals that the NA-related DEPs demonstrate a higher level of enrichment in the biological process of hydrogen peroxide metabolic process and shows the ability of activity as a calcium-dependent cysteine-type endopeptidase and specific binding to cadherins, with protein domain primarily being calcium proteinase III, which may potentially contribute to the superior therapeutic effects observed in NA. The EGb-related DEPs mainly have the ability to bind to actin filaments, which is different from NA. Therefore, the application of whole blood proteomics technology facilitates to distinguish the efficacy of drugs between different drug treatment groups.

However, limitations include technical challenges in whole-blood proteomics, such as the complexity and dynamic range of the blood proteome, which can hinder the detection of low-abundance proteins. In response to this, we have refined the sample processing protocols and enhanced the detection conditions. Additionally, variability in animal models, due to factors like genetic differences and physiological status, may affect the generalizability of the results to human conditions. Addressing these challenges in future studies will be essential to validate and translate our findings into clinical applications.

In summary, this study utilized whole blood proteomics technology to investigate the therapeutic effects of NA on cerebral ischemia-reperfusion injury. The results revealed that oxidative stress, Immune regulation, Cytoskeletal structure regulation and cell adhesion are the primary biological processes involved in this injury. Oxidative stress reaction and calcium-dependent adhesion may potentially be the most influential biological processes after the NA intervention. ENO1, STAT3, NME2, VCL, and CCT3 may serve as critical targets for therapeutic intervention this disease. The research provides a novel perspective and methodology to better understand the mechanisms of cerebral ischemia-reperfusion injury and develop innovative pharmacological treatments. The data demonstrate that integrating proteins from plasma, serum, and blood cells in whole blood proteomics methodology enables dynamic monitoring of protein network interactions, offering a comprehensive insight into biomarker discovery and exploration of mechanisms for complex brain vascular diseases.

## Data Availability

The mass spectrometry proteomics data have been deposited to the iProX (https://www.iprox.cn/page/project.html?id=IPX0010707000) and ProteomeXchange Consortium (https://proteomecentral.proteomexchange.org) via the iProX partner repository with the dataset identifier IPX0010707000 and PXD059362.
